# Implication of C-type natriuretic peptide derived from vascular endothelial cells on blood pressure regulation

**DOI:** 10.1186/2050-6511-16-S1-A28

**Published:** 2015-09-02

**Authors:** Kazuwa Nakao, Kazuhiro Nakao, Koichiro Kuwahara, Kenji Kangawa

**Affiliations:** 1 Medical Innovation Center, Kyoto University Graduate School of Medicine, Kyoto, Japan; 2 National Cerebral Cardiovascular Center, Kyoto, Japan; 3 Department of Cardiovascular Medicine, Kyoto University Graduate School of Medicine, Kyoto, Japan

## Clinical background

We previously reported the secretion of C-type natriuretic peptide (CNP) from vascular endothelial cells and proposed the existence of vascular natriuretic peptide system composed of endothelial CNP and vascular smooth muscle guanylyl cyclase-B (GC-B), the selective receptor for CNP, and implicated in the regulation of vascular tone, remodeling and regeneration [1-5]. However the functional role of the natriuretic peptide system is still unclear, because markedly short stature of CNP or GC-B knockout mice could be hardly studied for blood pressure regulation [6].

In the present study, we have assessed the functional significance of this system in the regulation of blood pressure in vivo using vascular endothelial cell-specific CNP knockout (CNP ecKO) and vascular smooth muscle cell-specific GC-B knockout (GC-B smcKO) mice generated using the Cre/loxP system. These mice showed neither the skeletal abnormality nor the early mortality observed in systemic CNP or GC-B knockout mice [6]. CNP ecKO mice exhibited significantly increased blood pressure and an enhanced acute hypertensive response to nitric oxide synthetase inhibition by NG-nitro-L-arginine methyl ester (L-NAME). In addition, acetylcholine-induced, endothelium-dependent vasorelaxation was impaired in rings of mesenteric artery isolated from CNP ecKO mice. Pulmonary vascular endothelial cells from CNP ecKO mice showed enhanced expression of genes encoding endothelin-1 (ET-1). In contrast, GC-B smcKO mice exhibited blood pressure similar to control mice, and acetylcholine-induced vasorelaxation was preserved in their isolated mesenteric arteries. Nonetheless, CNP-induced acute vasorelaxation was nearly completely abolished in mesenteric arteries from GC-B smcKO mice.

## Conclusion

These results demonstrate that endothelium-derived CNP contributes to the chronic regulation of vascular tone and systemic blood pressure by maintaining endothelial function independently of vascular smooth muscle GC-B and indicate the implication of the vascular natriuretic peptide system in blood pressure control.
